# Interspecies distribution and processing stability of ergothioneine and histidine in commercially available mushrooms

**DOI:** 10.17912/micropub.biology.002257

**Published:** 2026-07-22

**Authors:** Caden Guthrie, Semir Hasic, Harley Gordon

**Affiliations:** 1 Department of Biology, University of the Fraser Valley, Abbotsford, BC, Canada

## Abstract

Ergothioneine is a widespread thiol metabolite present in fungal and bacterial species. Ergothioneine has been demonstrated to increase the lifespan of the model laboratory species C. elegans and M. musculus through increased protein crosslinking. It is rapidly gaining popularity as a fungal derived nutraceutical compound, however the complete mushrooms species distribution and ecological role of ergothioneine is unknown. We show ergothioneine concentrations appear to be highest in mushroom species that are capable of cellulose degradation. We also show that ergothioneine is a thermally stable compound and does not appear to degrade but rather increases in concentration in cooked mushrooms.

**Figure 1. Ergothioneine and Histidine concentrations in Oyster and Shiitake mushrooms:** Figure 1. **A** : Moisture content of fresh, autoclaved and fried mushrooms samples. Solid columns are Oyster mushrooms and hatched columns are Shiitake mushrooms. Moisture content determined from 50.39 g of fresh Oyster mushrooms, 18.21 g of Fresh Shiitake mushrooms, 3.63 g of autoclaved Oyster mushrooms, 4.49 g of autoclaved Shiitake mushrooms, 1.34 g of fried Oyster mushrooms, and 2.65 g of fried Shiitake mushrooms; **B** : Histidine and Ergothioneine concentration scatterplots from individual samples (n =56); **C** : Histidine concentrations in fresh and cooked Oyster mushroom samples ; **D** : Histidine concentrations in fresh and cooked Shiitake mushroom samples; **E** : Ergothioneine concentrations in fresh and cooked Oyster mushroom samples; **F** : Ergothioneine concentrations in fresh and cooked Shiitake mushroom samples. Experimental replicates: fresh n=3, fried n=3, autoclaved n=3. Statistics: *** = p value < 0.01; * = p value < 0.05; ns = p value > 0.05. Table 1: Histidine and Ergothioneine concentrations in diverse fungal samples. *^Δ^ The mushroom species colonized by H. lactiflourum was not identified. H. lactiflourium colonize a range of mushrooms in the Russula, Lactifluus, and Lactarius genera. ^t^ n=3, ^s^ n=6). BLQ: below limit of quantitation.
*

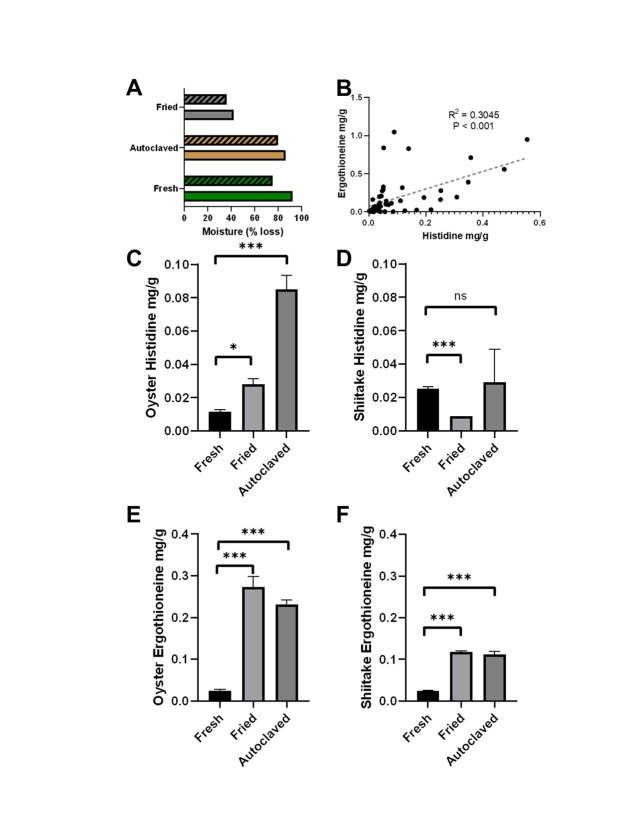



**Table:** 

Species	Phylum; Family; Common Name	Sample Type	Histidine mg/g Average(±SE)	Sample Type	Ergothioneine mg/g Average(±SE)
Agaricus bisporus	Basidiomycete	Fresh:	0.024 (±0.008)t	Fresh:	0.0002 (±0.00003) t
	Agaricaceae	Dried:	0.17 (±0.027) t	Dried:	0.019(±0.004) t
	Button Mushroom	Canned:	0.002 (±0.0001) t	Canned:	0.007 (±0.0004) t
Boletus edulis	Basidiomycete	Dried:	0.094(±0.025) t	Dried:	0.904 (±0.071) t
	Boletaceae				
	Porcini				
Cantharellus cibarius	Basidiomycete	Dried:	0.003 (±0.001) t	Dried:	0.002 – (one replicate detection)
	Cantharellaceae				
	Golden Chanterelle				
Hericium erinaceus	Basidiomycete	Dried Powder:	0.178 (±0.042)s	Dried Powder:	0.143(±0.01) s
	Hericiaceae	Capsule Powder:	0.051 (±0.021) t	Capsule Powder:	BLQ t
	Lions Mane				
Hypomyces lactifluorumΔ	Ascomycete	Dried:	0.025 (±0.004) s	Dried:	0.039 (±0.012) s
	Hypocreaceae				
	Lobster Mushroom				
Lentinula edodes	Basidiomycete	Fresh:	0.026 (±0.001) t	Fresh:	0.024 (±0.002) t
	Omphalotaceae	Dried:	0.048 (±0.0145) s	Dried:	0.171 (±0.037) s
	Shiitake				
Morchella esculenta	Ascomycete	Dried:	0.051 (±0.003) s	Dried:	0.207 (±0.041) s
	Morchellaceae				
	Yellow Morel				
Pleurotus ostreatus	Basidiomycete	Fresh:	0.012 (±0.001) t	Fresh:	0.025 (±0.004) t
	Pleurotaceae	Dried:	0.363 (±0.055) s	Dried:	0.511(±0.117) s
	Oyster Mushroom				
Tricholoma matsutake	Basidiomycete	Dried:	0.035(±0.002) t	Dried:	BLQ t
	Tricholomataceae				
	Matsutake				

## Description

Many fungal and bacterial species produce a histidine derived small sulphur containing metabolite called Ergothioneine (Beelman et al., 2020). Ergothioneine has potential nutraceutical properties as an antioxidant and an anti-aging compound (Wijeskara and Xu, 2024). Concentrated ergothioneine applications to *Mus musculus* and *Caenorhabditis***** *elegans* corresponded with a minor increase in average lifespan. This process is mediated through an identified molecular protein target, glycerol 3-phosphate dehydrogenase (GPDH). Ergothioneine can stimulate hydrogen sulphide production, and through GPDH increase protein crosslinking under high-stress conditions, which in turn may increase animal lifespan (Petrovic et al., 2025). Ergothioneine is a potent antioxidant, and one hypothesized to function in fungi. Glutathione is a separate sulphur containing metabolite known in fungi and capable of mitigating oxidative stress damage (Maras et al., 2014). Direct demonstration of ergothioneines proposed role in preventing oxidation reactions *in vivo* demonstration is lacking (Borodina et al., 2020). Given the diverse phyla Ergothioneine is present in, the biological role of this molecule may have organism dependant ecological functions (Sato et al., 2025). When consumed, humans uptake ergothioneine through an OCTN1 transporter (Alam et al., 2022). Ergothioneine has been shown to be protective of neurological and vascular systems, as it can cross the blood brain barrier, potentially reducing oxidative stress and inflammation in the brain (Beelman et al., 2020). Ergothioneine has been shown to lower risk of cardiometabolic disease when present at higher circulating concentrations, highlighting its potential role in long term health protection (Borodina et al., 2020; Cheah et al., 2021). As humans are unable to synthesize ergothioneine, the nutritional and functional value of mushrooms is closely tied to both their ergothioneine content and the extent to which it is retained following culinary processing (Beelman et al., 2020; Kalaras et al., 2017).

Ergothioneine in Aspergillus is synthesized from the The *egt* biosynthetic gene cluster which includes a cascade of enzymes encoded by *egtD* , *egtB* , *egtC* , and *egtE* in bacteria and *Egt1* / *Egt2* in fungi. Ergothioneine containing Cyanobacteria use a distinct, but convergent biosynthetic pathway (Liao et al., 2017). The fungal biosynthetic route starts with methylating histidine to produce hercynine, followed by the enzymatic sulfur insertion from cysteine that forms the final thiol containing ergothioneine molecule (Alam et al., 2022). Histidine and cysteine are molecular precursors for the biosynthesis of ergothioneine. We observed a moderate correlation of 0.3045 between histidine and ergothioneine concentrations ( Fig. 1B ), however while all samples had detectable concentrations of histidine, 10 out of 56 individual samples did not contain detectable ergothioneine. Therefore, we demonstrate that although histidine analysis may be somewhat predictive of ergothioneine concentration, it is not a reliable analytical predictor.

We assessed the distribution of ergothioneine in commercial mushroom and mushroom products from a broad assortment of mushroom species, we also monitored the concentration of histidine to determine if cooking impacts histidine as it does ergothioneine (Table 1). The tested mushrooms were predominantly dry, however some of which were fresh, canned, or further processed into powders and capsules. Fresh mushroom samples were processed in-house under defined procedures, however commercial drying, canning, and powdering conditions were unknown. These commercial processing conditions may impact ergothioneine or histidine concentrations (Lee et al., 2019). Mushroom products were quantified using liquid chromatography / tandem mass spectrometry for histidine and ergothioneine concentrations. Our results expand the species distribution examined by Dubost et al. (2006), while supporting their original findings. Dubost et al. (2006) identified the lowest concentration of ergothioneine in in *A. bisporus* (Button mushrooms or Cremini), while *L. edodes* (Shiitake), *P. ostreatus* (Oyster), and *B. edulis* (Porcini) contained the highest concentrations of ergothioneine. This result is consistent with ergothioneine analysis in mushrooms (Kalaras et al., 2017; Niu et al., 2020; Harasym et al., 2025). We have identified that the commercial mushrooms *C. cibarius* (Chantarelles), *H. lactifluorum* (Lobster), and *T. matsutake* (Pine/Matsutake) contain ergothioneine concentrations comparable or below *A. bisporus* mushrooms. Two samples of *H. erincius* (Lions Mane) mushroom powders were examined. One dried powder contained low concentrations of ergothioneine while gel capsules labelled as "Lions Mane” extracts contained no detectable ergothioneine. Interestingly, the wild mushroom *M. esculanta* (Morel), which is one of the few commercially collected ascomycetes, contained concentrations of ergothioneine comparable to *L. edodes* . The broad species distribution of ergothioneine concentration raises questions about the biological function of this small metabolite. Although ergothioneine is recognized as an antioxidant due to activity with 1,1-diphenyl-2-picrylhydrazil (DPPH assays) (Nguyen et al., 2023). that may not be the exclusive biological role of this compound. We note that in mushroom species examined, it is absent or low in concentration in species that are obligate ectomycorrhizal (Chantrelle and Pine) and absent or low in concentration in non-cellulose degrading saprophytes (Lobster and Button). The most abundant concentrations were found in Oyster, Shiitake, Morel, and Porcini mushrooms. Oyster and Shiitake mushrooms are saprophytic for cellulose, whereas Morel and Porcini mushrooms can be both mycorrhizal and/or saprophytic depending on environmental conditions (Bonito et al., 2025). Additional field collections and experimental assessments are required to determine if a cellulose saprophytic lifestyle correlates with ergothioneine concentrations.

Several studies indicate that ergothioneine is remarkably stable to heat and pH, yet its measured concentration in mushrooms may decrease depending on the cooking method and extent of leaching into water (Lo et al., 2020). For example, a study on shiitake mushrooms found that boiling mushrooms reduces ergothioneine content while roasting resulted in a slight increase (Lee et al., 2019). Our analysis found that both frying and autoclaving fresh weight Shiitake and Oyster mushrooms increased the concentration of ergothioneine significantly, supporting the claim the compound is exceptionally stable with cooking. Interestingly, the concentration of ergothioneine increased at a greater rate than the relative water loss due to frying. Figure 1A shows the moisture content of fresh and autoclaved samples are nearly equivalent, therefore the increase in Ergothioneine concentration is not due to moisture loss. In the fried samples approximately 40% moisture remained in the samples however in fried Oyster mushrooms ergothioneine concentration increased 10x, as opposed to the expected 2-3x increase due to moisture loss alone. Therefore, cooking may release ergothioneine molecules that are not released during an acidic methanol extraction. Ergothioneine may exist in complexed to cellular components that requires harsher processing conditions to release the molecules. We demonstrate that acidified methanol extractable ergothioneine concentrations increase with cooking, we postulate that the bioavailability of ergothioneine in Oyster and Shiitake mushrooms may increase following thermal processing.

Mushrooms are widely consumed by humans for nutritional and nutraceutical properties. The mushrooms themselves and the fungi they grow from occupy diverse and essential ecological roles. Ergothioneine is a small sulphur containing metabolite present in a variety of mushroom species, and may be associated with their ecological niche, as high concentrations are not ubiquitous across fungal families. Further research into the growth rates of a model mushrooms species, such as oyster mushrooms with varieties lacking ergothioneine may provide further insight into the ecological role of this molecule. Furthermore, ergothioneine is a thermally stable small molecule, with detectable concentrations increasing in thermally processed shiitake and oyster mushrooms. Therefore, individuals seeking to maximize ergothioneine consumption for presumed nutraceutical properties should fry or pressure cook their mushrooms. Highly processed and products containing dilute mushroom extracts such as Lions Mane tablets (Table 1) may contain less ergothioneine than whole mushrooms.

## Methods

*Samples and Metabolite extraction:*

Mushroom and mushroom products were selected from a range of commercially available sources: Lions Mane powder blend, OM, lot: 417710B192 ; Lions Mane capsules, Supplier: Purica, lot: P04900; 417710B192; Chantarelles (dried), Supplier: Ponderosa Mushrooms, lot: 2204904; Morel (dried), Supplier: W.C. wild foods, lot: 2825043833; Wild Porcini (dried), Supplier: Untamed Feast, lot: 00662109; Pine (dried), Supplier: W. C wild foods, lot: 2304102; Lobster (dried), Supplier: W.C. wild foods, lot: 2203108;Oyster mushrooms (Fresh, dried in house), Supplier: Compliments organic, lot: unidentified; Shiitake (fresh and dried in house), Supplier: Choices Market, lot: unidentified; Button (fresh and dried in house), Supplier: Choices Market, lot: unidentified; Button (canned), Money’s, lot: not recorded. Samples dried in-house for metabolite analysis were cut into 2 cm strips and dried for 25 h at 50º C. For metabolite extraction approximately 10 mg of mushroom sample was macerated with a pestle in 1 mL of 50% H _2_ O:50% Methanol and 0.1% Formic acid. Samples were vortexed for 30 s and sonicated in a bath sonicator for two minutes. Samples were then centrifuged for 2 min at 10,000 rcf. The supernatant was removed and filtered with a 0.22 µm PTFE filter and stored in a glass vial at -20º C prior to analysis. Standards and processing chemicals were purchased from Millipore-Sigma (MilliporeSigma Canada Ltd, Oakville, ON, Canada). To simulate a pressure-cooking process and examine the impact on ergothioneine, fresh frozen Oyster and shiitake mushroom samples were autoclaved for 20 minutes at 15 PSI and 120º C. For fried samples, fresh frozen mushrooms were added to a hot pan containing a small volume of avocado oil. Samples were fried for 10 - 12 minutes until they browned and appeared cooked. Fried samples were blotted to remove excess oil and processed for metabolite extraction. Moisture loss was determined by weight loss in samples following 16 h drying at 50ºC.

*Quantification Procedure:*

Histidine and ergothioneine were quantified on a 1290 infinity ii LC system coupled to an Ultivo triple quadrupole mass spectrometer (LC-MS/MS) (Agilent Technologies Inc, Santa Clara, CA, USA). 10 µL of filtered extract was injected and separated on a Zorbax SB-C18 (3.0 mm x 0.5 mm x 1.8 µm) guard column and separated with a Zorbax SB-C18 (2.1 mm x 50 mm x 1.8 µm) column at 40ºC. The solvent flow rate was constant at 0.3 mL/min. The solvents used were A: deionized water with 0.1% Formic acid, and B: acetonitrile. Solvent gradients were 0.0– 1.0 min, hold 95% A; 1.0 – 5.0 min gradient 95% A to 5% A and 95% B; 5.0– 7.0 min hold 95% B; 7.0 – 8.0 min gradient 5% A to 95% A; 8.0-9.0 min hold 95% A. Samples were ionized using an Agilent AJS electrospray ionization source in positive mode. The source parameters were gas temperature 300 ºC, gas flow 8.0 L/min, nebulizer pressure 35 psi, sheath gas temperature 180 ºC, sheath gas flow 10 L/min, capillary voltage 4000 V, and nozzle voltage 1500 V. The quantitative ion transition for ergothioneine was 230>127 at a collision energy (CE) of 20 V, the qualitative ion was 230>60 at CE 15 V. For histidine the quantitative ion transition was 156>110 at CE 17 V and the qualifying ion transition was 156>83 at CE 25 V. Samples were quantified by comparison to regression of known concentrations, and with matching qualifying ion ratios in Agilent Quantitative QQQ software (Agilent Technologies Inc). Standard concentrations ranged from 10 µg/mL to 1 ng/mL. Statistical comparisons of cooked samples were students t-tests conducted in Graphpad Prism.
